# The International Conference on Intelligent Biology and Medicine (ICIBM) 2018: bioinformatics towards translational applications

**DOI:** 10.1186/s12859-018-2460-3

**Published:** 2018-12-28

**Authors:** Xiaoming Liu, Lei Xie, Zhijin Wu, Kai Wang, Zhongming Zhao, Jianhua Ruan, Degui Zhi

**Affiliations:** 1 Human Genetics Center, School of Public Health, The University of Texas Health Science Center at Houston, Houston, TX 77030 USA; 2 Present address: College of Public Health, University of South Florida, Tampa, FL 33612 USA; 3 Department of Computer Science, Hunter College & The Graduate Center, The City University of New York, New York, NY 10065 USA; 4 Department of Biostatistics, Brown University, Providence, RI 02912 USA; 5 Raymond G. Perelman Center for Cellular and Molecular Therapeutics, Children’s Hospital of Philadelphia, Philadelphia, PA 19104 USA; 6 Department of Pathology and Laboratory Medicine, University of Pennsylvania, Philadelphia, PA 19104 USA; 7 Center for Precision Health, School of Biomedical Informatics, The University of Texas Health Science Center at Houston, Houston, TX 77030 USA; 8 Department of Computer Science, The University of Texas at San Antonio, San Antonio, TX 78249 USA

## Abstract

The 2018 International Conference on Intelligent Biology and Medicine (ICIBM 2018) was held on June 10–12, 2018, in Los Angeles, California, USA. The conference consisted of a total of eleven scientific sessions, four tutorials, one poster session, four keynote talks and four eminent scholar talks, which covered a wild range of aspects of bioinformatics, medical informatics, systems biology and intelligent computing. Here, we summarize nine research articles selected for publishing in *BMC Bioinformatics*.

## Introduction

The 2018 International Conference on Intelligent Biology and Medicine (ICIBM 2016) provided a multidisciplinary forum for computational scientists and experimental biologists to share their most recent findings in the field of cancer genomics, systems biology, medical informatics, big data analytics and machine learning, among others. The conference was held on June 10–12, 2018, in Los Angeles, California, USA. More than 160 researchers and students across the world attended the meeting. In this special issue, we have collected nine original research articles reflecting the cutting edge researches in bioinformatics. As the advance of all kinds of omics studies, bioinformatics has beome the indispensable powerhouse behinds all analyses. This is reflected in our selection, as these papers cover traditional areas in genomics, transcriptomics, proteomics, and literature mining, as well as new research foci such as Hi-C data and electronic health record. We also observe a shift of research interest from developing tools for analyzing high-throughput data towards translational applications. This trend is also evident in the selection as majority of the studies have a broad goal of better understanding human diseases. In the following, we briefly summarize the nine selected papers.

### The science program for the ICIBM 2018 bioinformatics track

In the first paper, He et al. [1] developed an innovative semi-parametric latent variable differential network model for investigating the structural difference of genetic networks under two experimental conditions, such as two gene expression data sets. The advantages of this new model include the capability of handling complex biological data with various types (discrete or continuous) and relaxing normality assumption that often does not hold in the real data. Theoretical analysis demonstrated that the new methods achieve the same parametric convergence rate for both the differential structure recovery and difference of the precision matrices estimation. Numerical simulation and real application also showed the advantages of the new model as to providing deeper understanding of the mechanism of diseases.

Top-down mass spectrometry performs particularly well in identifying proteoforms with multiple modifications and/or alterations. When applying this technology to a species that does not have a reference protein sequence database for proteoform identification, a homologous protein sequence databased can be used as an alternative. Li et al. [2] evaluated the performance of TopPIC, a commonly used software for top-down mass spectral identification, on top-down mass spectral identification with homologous protein sequences. A *Escherichia coli* K12 MG1655 and a human MCF-7 cells top-down mass spectrometry data sets were used in the evaluation. For each data set, the mass spectra were searched separately against a reference proteome database and a homologous proteome database. The results showed that TopPIC is able to identify many proteoform spectrum matches and localize unknown alterations using homologous protein sequences with no more than 2 mutations.

In the third paper, Shen [3] reported DLAD4U (Disease List Automatically Derived For You), a new web-based disease retrieval and prioritization tool based on PubMed literature. It utilizes existing resources of the NCBI to achieve computational efficiency and statistical analyses to ensure accuracy. Easy usage and interpretation of the results is achieved via a simple Google-like interface. Using selected genes and drugs as query terms and manually curated data as “gold standard”, the authors demonstrated the superior performance of DLAD4U compared to other disease search engines.

In the next paper, Liu and Wang [4] addressed one of the key issues of using Hi-C data: the unclear relationship between spatial distance and the number of Hi-C contacts. This relationship is essential for understanding some significant biological functions, such as the enhancer-promoter interactions. The authors proposed a new method for inferring the converting parameter and the pairwise Euclidean distances based on the topology of Hi-C complex network (HiCNet). The inferred distances had a higher correlation with fluorescence in situ hybridization (FISH) data, fitted the localization patterns of Xist transcripts on DNA, and better matched 156 pairs of protein-enabled long-range chromatin interactions detected by ChIA-PET. A 40 kb high-resolution 3D chromosomal structures of mouse male ES cells were then reconstructed using the new method.

One of the consistent challenges in precision medicine is to accurately predict the sensitivity of a tumor to an anti-cancer compound. Large-scale pharmacogenomics studies, like CCLE and GDSC, hold the promise for designing an accurate prediction model. However, integrating information from multiple resources faces the challenge of removing the distribution shift between data. Dhruba et al. [5] proposed to use transfer learning methodologies to eliminate this distribution shift and design effective drug sensitivity prediction models in a target database by incorporating data from a secondary database. More specifically, the authors presented two novel approaches based on latent variable cost optimization and polynomial mapping. With different scenarios, they demonstrated that the proposed approaches accomplish a better prediction of drug sensitivities compared to database-specific individual models and existing transfer learning approaches, with the nonlinear mapping model exhibits the best overall performance.

Identifying local recurrences in breast cancer patients is important for clinical research and practice. Zeng et al. [6] proposed a novel concept-based filter and a prediction model to detect local recurrences using electronic health records (EHR) of breast cancer patients. Unlike typical clinical NLP (natural language processing) systems, the authors proposed to utilize a positive set of concepts related to breast cancer local recurrence using MetaMap, a tool for identifying medical concepts in text. The new model was compared with three baseline classifiers using either full MetaMap concepts, filtered MetaMap concepts, or bag of words. The results showed that the new model achieved the best performance and provided an automated and effective way to identify breast cancer local recurrences.

Chowdhury et al. [7] proposed another new method for analyzing electronic medical record (EMR). The new method is targeting a challenge in Entity Recognition (NER), a sub-field of information extraction aimed at identifying specific entity terms such as disease, test, symptom, genes etc., in the situation when the available EMR is limited. The authors proposed a multitask bi-directional RNN model as a potential solution of data augmentation to enhance NER performance with limited data. The evaluation test showed the superior performance of the proposed model compared to the baseline model in terms of micro average F-score, macro average F-score and accuracy.

Studying disease-disease relationships has wide applications in biomedical field, such as understanding disease mechanism and drug discovery. The FDA Adverse Event Reporting System (FAERS) contains rich information about patient diseases, medications, drug adverse events etc. Zheng and Xu [8] systematically explored this data resource to construct a disease comorbidity network (DCN) with 1,059 disease nodes and 12,608 edges using association rule mining (14,157 rules). The DCN shows good performance in capturing known disease comorbidities and is well correlated with disease semantic similarity, disease genetics and disease treatment. Using asthma as a case study, the authors also demonstrated that the DCN has potential in uncovering novel disease relationships.

In the last paper, Khan et al. [9] developed a computational tool, integrated Mental-disorder GEnome Score (iMEGES), to prioritize disease-relevant genes and variants with personal genomes. The new tool uses deep neural network approaches to integrate diverse sources of input information, including whole-genome variants and clinical phenotype terms of an individual with mental disorders, and outputs prioritized lists of variants and genes that may be relevant to the phenotypes. iMEGES was evaluated using multiple datasets of mental disorders, and achieved improved performance compared to competing approaches. The tool can be used in population studies for prioritizing novel genes or variants associated with disease susceptibility, as well as on individual patients for identifying genes or variants with large effect on mental disorders.
